# Le volvulus gastrique idiopathique aigu: à propos d'une nouvelle observation

**DOI:** 10.11604/pamj.2013.14.31.2374

**Published:** 2013-01-22

**Authors:** Mouhsine Abdelilah, Anzaoui Jihad, Bouchentouf Rachid

**Affiliations:** 1 Service de Radiologie, Hopital Militaire Avicenne, Marrakech, Maroc; 2 Service d'Urologie, Hôpital Militaire Avicenne, Marrakech, Maroc; 3 Service de Pneumologie, Hôpital Militaire Avicenne, Marrakech, Maroc

**Keywords:** Estomac, volvulus, tomodensitométrie, chirurgie, stomach, volvulus, tomodensitometry, surgery

## Abstract

Le volvulus gastrique est une rotation anormale de l'estomac autour de son axe. La forme aiguë constitue une urgence chirurgicale. Le diagnostic est souvent retardé en raison d'une symptomatologie fréquemment non spécifique. Des signes respiratoires tels la dyspnée et le hoquet peuvent révéler cette pathologie. Les auteurs rapportent une nouvelle observation de volvulus gastrique aigu chez un adolescent de 17 ans, diagnostiqué par la tomodensitométrie, et confirmé par une intervention chirurgicale. Le traitement est chirurgical et consiste à détordre et fixer l'estomac pour prévenir la récidive.

## Introduction

Le volvulus gastrique aigu est une urgence chirurgicale rare, réalisant une occlusion digestive haute par torsion de l'estomac d'au moins 180°. La Tomodensitométrie actuellement occupe une place importante dans le diagnostic positif. Son traitement est chirurgical, et le diagnostic souvent retardé en raison d'une symptomatologie fréquemment aspécifique.

## Patient et observation

Jeune patient de 17 ans sans antécédents pathologiques particuliers se plaignant depuis une semaine de dyspnée d'effort et de hoquet non amélioré par un traitement symptomatique. Admis au service des urgences pour un syndrome abdominal douloureux aigu atypique avec vomissement précoce sans arrêt des matières et des gaz. L'examen clinique trouve une distension abdominale et un abdomen sensible sans signe de péritonite. Le bilan biologique est normal.

**Figure 1:**
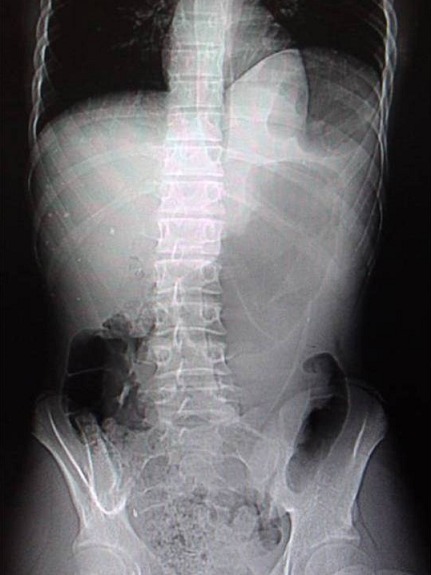
Le topogramme montre une ascension de l'hémicoupole diaphragmatique gauche, refoulée vers le haut par l'estomac

Le topogramme montrait une ascension de l'hémi coupole diaphragmatique gauche, refoulée vers le haut par l'estomac qui présentait une volumineuse poche à air et peu d'air intestinal (Figure 1).

**Figure 2:**
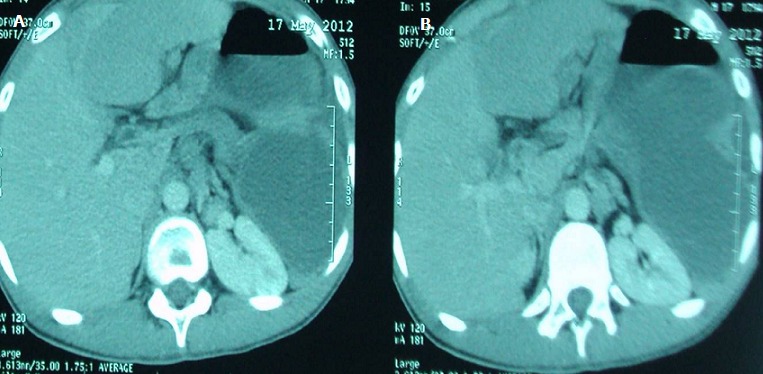
TDM réalisée en mode spiralé après injection de produit de contraste selon le plan axial (A) et la reconstruction coronale (B) confirme l'existence du volvulus gastrique et de la présence d'une rate « baladeuse » et une importante ascension du rein gauche

**Figure 3:**
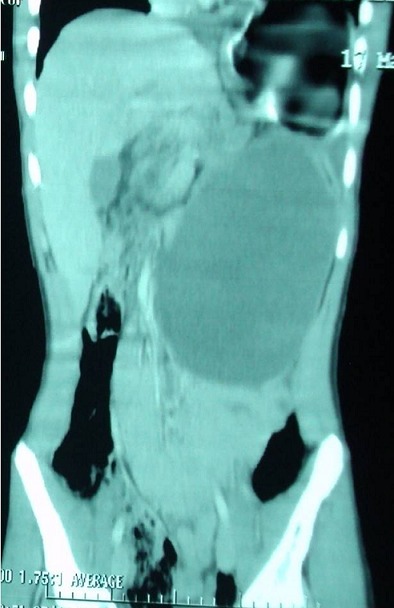
TDM abdominale:reconstruction coronale

La TDM confirme l'existence d'une dilatation hydro-aérique majeure de l'estomac, et révèle des signes évocateurs de torsion, avec une rate « baladeuse » sous hépatique, une importante ascension du rein gauche (Figure 2, Figure 3).

**Figure 4:**
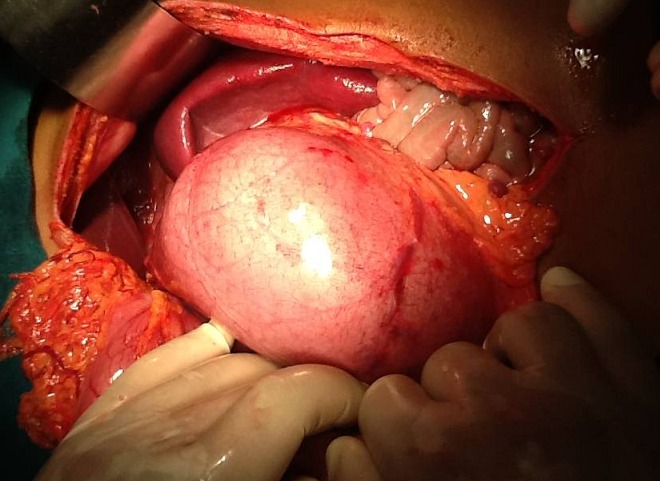
Aspect per opératoire montrant le volvulus gastrique organoaxial

L'intervention chirurgicale confirme le diagnostic de volvulus gastrique type oganoaxial, lié à une absence des différents moyens de fixation de l'estomac hormis le ligament gastrophrénique relâché, rate baladeuse et une importante hypoplasie de la coupole diaphragmatique gauche (Figure 4), et en permet le traitement.

Les suites postopératoires ont été simples, aussi bien dans l'immédiat qu'aux contrôles ultérieurs.

## Discussion

Le volvulus gastrique est une urgence abdominale rare chez l'enfant [1–3]. Sa fréquence est probablement sous estimé car des formes spontanément résolutives sont possibles [4]. Il est secondairement associé à des défauts congénitaux prédisposants telle une laxité des moyens de fixation de l'estomac comme ce qui a été noté dans notre cas, où à des anomalies congénitales ou acquises diaphragmatiques.

D'autres anomalies abdominales, spléniques et surtout hépatiques sont souvent associées au volvulus gastriques [4–6]. Dans notre cas la rate était en prévertébrale sous le foie gauche sans moyens de fixation notable notamment pas de ligament gastrosplénique (rate baladeuse appelée également oscillante “Wandering Spleen”.

La forme primaire est observée dans 30% des cas. Il peut être secondaire à d'autres affections comme une hernie hiatale ou diaphragmatique, un traumatisme abdominal, une asplénie et une gastroplastie [7].

4 formes anatomiques de torsion gastriques peuvent être rapportées, dont deux sont principales, le première est dite organoaxiale, sa rotation s'effectue autour de l'axe cardiopyloriques comme celle de notre cas réalisant un vrai volvulus. La deuxième forme plus fréquente appelée mésentéricoaxiale, sa rotation s'effectue suivant un axe longitudinal du petit épiploon [8, 9]. Une forme mixte à été décrite, et une forme dite inclassable [10].

La clinique n'est pas spécifique, souvent une occlusion ou une douleur abdominale comme chez notre cas. La triade de Borchardt est évocatrice, associant une douleur épigastrique majeure avec irradiations vers le dos et/ou l'hypochondre ou l'hémithorax gauche, efforts de vomissements inefficaces, intolérance alimentaire absolue avec difficultés ou impossibilité de mise en place d'une sonde nasogastrique [1, 11].

L'Abdomen sans préparation (ASP) est habituellement peu contributif, il peut montrer une distension gazeuse de la partie haute de l'abdomen, des niveaux hydroaériques rétrocardiaques en cas d'hernie hiatale associée, et parfois un emphysème de la paroi gastrique [4]. Les examens d'opacification digestive sont spécifiques mais souvent de réalisation difficile [4].

La Tomodensitométrie actuellement occupe une place importante dans le diagnostic positif, grâce aux reformations multi planaires [4], son aspect peut varier selon le degré et les points de torsion, elle est utile à la fois pour reconnaitre la torsion de l'estomac, éliminer une autre pathologie abdominale, elle permet aussi de guider un éventuel geste chirurgical selon la gravité de l'état clinique.

L'aspect évoquant une torsion de l'estomac associé une distension hydroaèrique gastrique marquée et une zone d'épaississement tissulaire, avec congestion vasculaire, séparant un contingent gastrique purement aérique et un autre contingent hydrique, qui était traversée par la sonde nasogastrique, dont le trajet est bien suivi sur les niveaux de coupes successifs [4, 12].

Les complications sont la nécrose gastrique ou la péritonite aigue par perforation gastrique en péritoine libre. Le traitement est toujours chirurgical, il s'impose en urgence dés qu'on a un diagnostic posé ou suspecté. Il a pour but de détorde l'estomac et de réaliser ou non une gastropexie [1, 4].

Actuellement, on recourt de plus en plus à la technique coelioscopique qui permet en même temps de faire le diagnostic et de traiter la pathologie [1, 2, 4].

## Conclusion

Le volvulus gastrique est une affection rare et constitue une vraie urgence chirurgicale. Son diagnostic peut être porté grâce à la TDM, qui doit, actuellement, être l'examen d'imagerie réalisé en première intention lorsque cette pathologie est évoquée. Une prise en charge précoce amène une évolution habituellement favorable.
